# Flexible nanosheets for plasmonic photocatalysis: microwave-assisted organic synthesis of Ni–NiO@Ni_2_CO_3_(OH)_2_ core–shell@sheet hybrid nanostructures†

**DOI:** 10.1039/d3na00583f

**Published:** 2023-11-03

**Authors:** Ekta Rani, Parisa Talebi, Terhi Pulkkinen, Vladimir Pankratov, Harishchandra Singh

**Affiliations:** a Nano and Molecular Systems Research Unit, University of Oulu FIN-90014 Finland Harishchandra.Singh@oulu.fi; b Institute of Solid-State Physics, University of Latvia 8 Kengaraga iela 1063 Riga Latvia

## Abstract

Visible light-active nickel-based plasmonic photocatalysts provide a cost-effective alternative to noble metals. However, their rarity, fragility, and limited understanding pose challenges. This work presents a microwave-assisted organic synthesis of a Ni–NiO@Ni_2_CO_3_(OH)_2_ core–shell@sheet plasmonic photocatalyst. By employing time and power dependent synthesis, this catalyst exhibits flexible Ni_2_CO_3_(OH)_2_ nanosheets enveloping the Ni–NiO structure, surpassing the pristine Ni@NiO/NiCO_3_ core–shell counterpart. Chemical reaction mechanisms suggest that irradiation of pristine Ni–NiO/NiCO_3_ nano structures leads to breakage of amorphous NiCO_3_ to Ni^2+^ and CO_3_^2−^, which further, in the presence of water solvent, interacts with OH^−^ ions leading to the formation of Ni(CO_3_)·Ni(OH)_2_. With enhanced light absorption and photocatalytic properties, the resulting core–shell@sheet photocatalyst demonstrates double the hydrogen evolution reaction yield (40 μmol g^−1^ h^−1^) compared to the pristine catalyst (20 μmol g^−1^ h^−1^). The enhanced H_2_ yield is attributed to the flexible sheets, cross-dimensional photocatalyst structure, increased surface area for surface reactions, and higher H_2_ activity of Ni_2_CO_3_(OH)_2_. This research showcases the potential of microwave-assisted synthesis in developing flexible nanosheets with superior solar water splitting performance.

## Abstract 2

Visible light-active nickel-based plasmonic photocatalysts provide a cost-effective alternative to noble metals.

## Graphical abstract

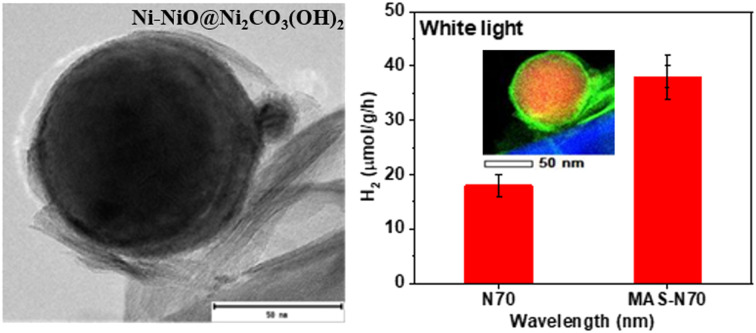

## 1. Introduction

The advancement of civilization hinges upon our capacity to discover, extract, and harness energy with ever-growing proficiency. This necessity has prompted a transition from non-renewable fossil fuels to renewable energy sources. In this context, the emerging hydrogen era represents a significant stride towards a sustainable future. The production of H_2_ from abundant H_2_O using inexhaustible solar energy is a viable option. However, despite intense research devoted to the exploration of efficient photocatalysts, the corresponding efficiency remains unsatisfactory for commercial applications.^1–3^ In order to enhance overall performance, researchers have directed their efforts towards the advancement of nanocomposite-based catalysts.^4^ Among diverse low-dimensional composites, 2D material-based nanocomposites have emerged as potential candidates for next-generation devices due to their exceptional mechanical strength, flexibility, large surface area, and rigidity, as well as their enhanced electrical and optical properties.^5^ In particular, unusually strong electronic coupling in these 2D nanocomposites can be achieved because of the exposed atoms on the interfacial surface of 2D materials. Thus, engineering of 2D nanocomposites with a lower production cost demands facile synthesis techniques. Among various techniques available, microwave-assisted synthesis is considered a facile synthesis method. It allows the possibility of performing reactions in a short time by direct interaction of microwave energy with the reaction mixture as opposed to the indirect transfer of energy by utilizing an oil bath.^6^ Moreover, water as solvent (or cosolvent) being cost effective, nontoxic, nonflammable, and environmentally benign provides opportunities for organic/green synthesis.

Recently, surface plasmon resonance (SPR)-based heterogenous photocatalysts have boosted the photocatalytic efficiency under visible light irradiation.^7–9^ The performance of plasmonic photocatalysis is intricately linked to the overall system, encompassing the specific metal or noble metal used, as well as the supporting materials (including size, shape, crystallinity, porosity, and contact form).^10,11^ Moreover, the addition of a ternary component/co-catalyst to such a binary plasmonic photocatalytic system further boosts the performance.^12^ These factors exert a profound influence on the selection of cheaper plasmonic metals and supports, which in turn affect the efficiency and effectiveness of plasmonic photocatalysis reactions. Thus, crucial considerations are required in optimizing chemical processes for the hydrogen evolution reaction (HER). In addition to costly noble metals, the utilization of cost-effective nickel (Ni) and Ni-based compounds exhibits immense potential. These compounds possess a remarkable ability to undergo multiple oxidation states, facilitating the formation of complex structures. This unique characteristic enables them to deliver enhanced performance in terms of catalytic activity, selectivity, and stability, thereby meeting the demanding requirements of various chemical processes. By leveraging the versatility of Ni and its compounds, researchers can unlock new avenues for achieving efficient and sustainable catalysis. For example, Ni@NiO core@shell nanostructures play a significant role in hydrogen generation^13,14^ over pure NiO, or mixed dispersion of NiO nanoparticles and Ni metal.^15^ Moreover, Ni nanostructures–Ni(OH)_2_ combination has also been reported for emerging energy and environmental applications.^16,17^ Recently, we have also employed pristine Ni–NiO/NiCO_3_ core–shell nanostructures and their vacuum annealed counterparts for the photocatalytic HER under visible light illumination. Another variation of Ni-based compounds involves Ni_2/3_(CO_3_)_2_(OH)_2/4_, which have been investigated as potential materials for faradaic capacitors due to the hydrophilic properties of (CO_3_)^2−^. This compound increases the wettability of the electrode surface and thus achieves higher capacitive efficiency and electrochemical characteristics than oxide/hydroxide-based materials. However, this has not been explored for the photocatalytic HER yet.

To enhance the yield of the hydrogen evolution reaction (HER), an experiment was conducted using microwave-assisted synthesis on pristine Ni–NiO/NiCO_3_ core–shell nanostructures. In this study, the initial core–shell nanostructures consisting of Ni–NiO/NiCO_3_ with an average size of approximately 70 nm were subjected to microwave synthesis, resulting in the formation of a hybrid nanostructure with cross-dimensional characteristics, comprising Ni–NiO core–shell and NiCO_3_·Ni(OH)_2_ nanosheets. Through detailed analysis of the structure, morphology, and spectroscopic properties, the growth mechanism of these cross-dimensional hybrid nanostructures was elucidated. Significantly, this study also provides novel insights into the physical nature of the HER mechanism and the role of nickel carbonate hydroxide in such photocatalytic systems, which have not been previously reported.

## 2. Experimental

### 2.1. Microwave assisted synthesis

Ni nanopowder (CAS no. 7440-02-0 (EINECS 231-111-4) A092, A095 and A096) of nominal purity of 99.8% with an average diameter of 70 nm referred to as N70 (pristine Ni–NiO/NiCO_3_ core–shell) was purchased from Hongwu International Group Ltd (HWNANO). The microwave-assisted organic synthesis was carried out in a domestic microwave oven (Panasonic NN-3496). The microwave-assisted synthesis was carried out as follows: 225 mg of N70 was added to 225 mL of Milli Q water and sonicated for 2 h at room temperature. The solution was divided into nine sets each containing 25 mL solution. Solutions were microwaved at three different powers of 100, 440, and 800 W. Moreover, different times of synthesis were taken into consideration: 30 s, 1, 5, 15, and 30 min. Rather than continuous exposure, solutions were subjected to microwave irradiation in a discrete step allowing air cooling of solution in-between. The pattern for air cooling samples was 1 min microwave and 2 min air cooling. To dry the as-prepared samples, the samples were heated to approximately 100 °C in ambient air by utilizing a hot plate. Dried samples were named MAS-N70 and kept in sample bottles as dry powder.

### 2.2. Characterization

X-ray diffraction (XRD) measurements were carried out by using a Rigaku Smart Lab equipped with a five-axis *θ*–*θ* goniometer and 1D solid-state detector and scintillator using Co-Kα (*λ* = 1.79 Å, 40 kV, and 135 mA) radiation. The field emission scanning electron microscope (SEM) images were collected on a Zeiss Sigma field emission SEM. Transmission electron microscopy (TEM), energy dispersive spectroscopy (EDS) mapping, and selective area electron diffraction (SAED) were performed using a JEOL JEM-2200FS EFTEM/STEM. Tomography measurements were also carried out from −60° to +60° with a 1° step size, giving rise to in total 121 images using a JEOL JEM-2200FS EFTEM/STEM. X-ray photoelectron spectroscopy (XPS) characterization was performed with Al-Kα using a Thermo Fisher Scientific ESCALAB 250Xi XPS System. Energy calibration of the XPS was performed by using the C 1s peak at 284.8 eV and Au is used as a sample holder for the XPS measurements. Raman spectroscopy measurements were carried out using a Thermo Scientific™ DXR2xi Raman Imaging Microscope. Brunauer–Emmett–Teller (BET) surface area measurements were carried out using a Micrometrics ASAP 2020 apparatus. UV-visible (UV-vis) absorbance spectra were obtained using a Shimadzu UV-2600 spectrophotometer in the absorption range of 300 to 700 nm. UV-vis absorbance was measured using a quartz cuvette with a volume of 3.5 mL. Luminescence and time-resolved luminescence measurements of optimized samples were carried out using a photoluminescence (PL) spectrometer FLS1000 (Edinburgh Instruments). The luminescence decay kinetics were recorded under the excitation of a 375 nm pulsed laser coupled with the spectrometer at room temperature.

### 2.3. Photocatalytic HER measurements

The photocatalytic hydrogen evolution activity of N70 (pristine) and MAS-N70 was measured using a quartz bottle with dimensions 90 mm × 35 mm (height × diameter) and a total volume of ∼60 mL. In a typical run, 5 mg of catalyst was suspended in 25 mL of deionized (DI) water followed by sonication for two minutes. LED light sources equipped with a magnetic stirrer in a Perfect Light PCX50B photo reactor were used for excitation. The white LED has a nominal power of 0.495 W. The solution was exposed to light for 2 h at room temperature. To measure the amount of produced H_2_, an Agilent 8860 gas chromatograph (GC) equipped with a hydrogen sensitive column was used. Calibrated microsyringes were used to inject samples. All measurements were carried out without any cocatalyst and electron/hole sacrificial agents.

## 3. Results and discussion

### 3.1. Formation of flexible nanosheets: morphological determination

**Fig. 1:**
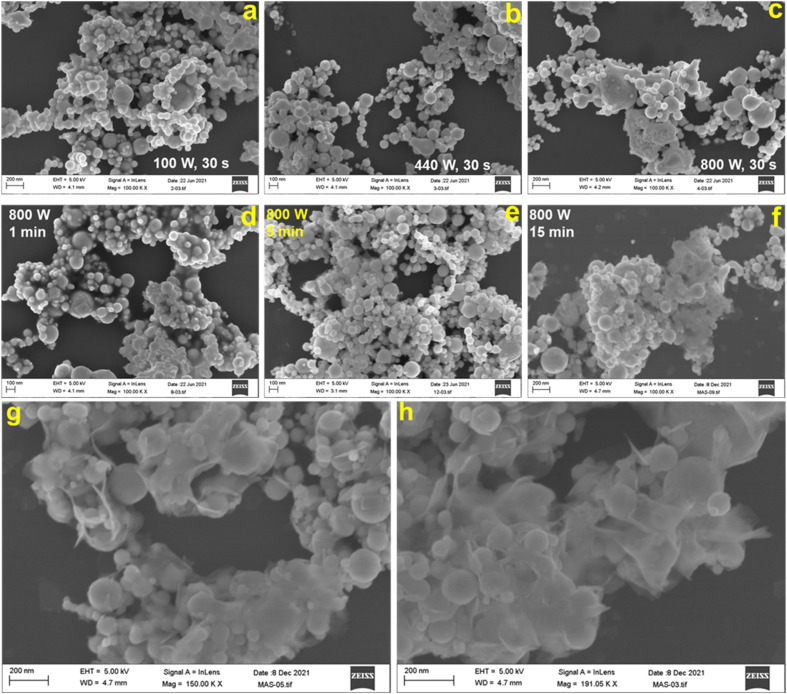
SEM image of N70 microwaved for 30 s (a) at 100 W, (b) at 440 W, and (c) 800 W. SEM image of N70 microwaved at 800 W for (d) 1 min, (e) 5 min, and (f) 15 min. High-resolution SEM image of MAS-N70 microwaved at 800 W for 30 min (g and h).

**Fig. 2:**
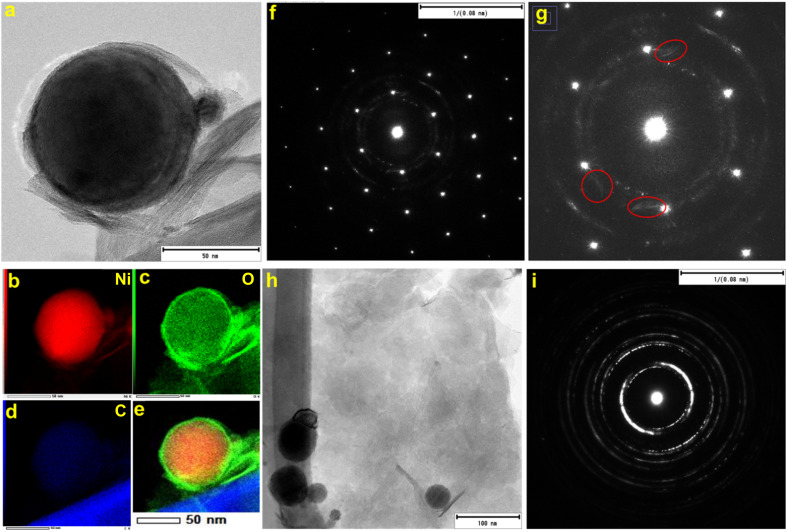
(a) TEM image, (b–e) EDS mapping, (f) corresponding SAED pattern, (g) zoomed-in region of the SAED pattern, (h) TEM image of nanosheets, and (i) corresponding SAED pattern.

Fig. 1 shows the representative field emission SEM images for N70 microwaved under different synthesis conditions. As shown in the images, irrespective of synthesis power, all samples microwaved at 30 s (Fig. 1a–c) and 1 s (Fig. 1d) showed a spherical morphology, which is the same as that of N70. An increase in the synthesis time to 5 min did not lead to change in the spherical morphology at 100 and 440 W. However, SEM images show that the spherical morphology starts showing change to a sheet morphology at 800 W (Fig. 1e). Furthermore, the sheet morphology is clearly evident in the sample microwaved at 800 W for 15 min (Fig. 1f). An increase in the synthesis time to 30 min led to an increase in the density of sheets in the sample (Fig. 1g–h). This shows that microwave irradiation of N70 leads to morphological transformation giving rise to nanosheet formation. Thus, the sample microwaved at 800 W for 30 min (referred to as MAS-N70) was further studied using TEM to get into the details of the morphology and structure. The TEM image in Fig. 2a and tomography measurements (Video 1†) confirm the overall morphology of the samples as spherical along with the presence of flexible nanosheets (NSs) surrounding the spherical nanoparticles (NPs), which agrees with the SEM results. The EDS mapping in Fig. 2b–e depicting the elemental distribution of N70 confirms the core–shell structure of spherical NPs along with the presence of Ni in the core and Ni and O in the shell, which agrees with our previous work on N70. Additionally, Ni, O and C are evident in NSs as well. As per our previous work, it is known that the core is purely made of Ni, whereas the shell consists of crystalline NiO and amorphous NiCO_3_. To get further details about NSs, SAED was carried out in two different regions: (i) in the region that was used to carry out TEM and EDS mapping (Fig. 2f and g) and (ii) in the region where mainly NSs are observed (Fig. 2h and i). Fig. 2f shows a [110] zone axis SAED pattern of MAS-N70 displaying well-defined diffraction spots corresponding to crystalline Ni NPs. In addition to diffraction spots, the SAED pattern displays (i) a polycrystalline ring shape and (ii) a moon shape (marked with red ovals in Fig. 2g: zoomed-in view of Fig. 2f). The observed set of rings with some spots depending on the crystallite sizes can be attributed to the nanocrystalline material. The calculated *d*-spacing of ∼2.33, 2.04, and 1.39 Å corresponds to the (003), (012), and (104) *hkl* planes of NiO, respectively. The observed moon shaped rings can be due to the flexible nature of NSs bent around spherical NPs. In order to understand the structure of these NSs, a region was chosen wherein only NSs are observed in TEM and SAED was carried out (Fig. 2f and g). The observed set of rings again suggest the polycrystallinity of NSs. Furthermore, the calculated *d*-spacing (Table 1) suggests that NSs can possibly be made of Ni(OH)_2_, Ni_2_CO_3_(OH)_2_ or Ni_3_CO_3_(OH)_4_, which needs to be further investigated.

**Table 1:** Calculated *d*-spacing for sheets and *d*-spacing for Ni(OH)_2_ (ICDD # 00-014-0117), Ni_2_CO_3_(OH)_2_ (ICDD # 00-035-0501), and Ni_3_CO_3_(OH)_4_ (ICDD # 00-016-0164)

S. no.	Nanosheets *d*, Å	Ni(OH)_2_*d*, Å (I)	Ni_2_CO_3_(OH)_2_*d*, Å (I)	Ni_3_CO_3_(OH)_4_*d*, Å (I)
1	2.6731	2.70 (45)	2.61 (100)	2.73 (50)
2	2.2834	2.33 (100)	2.19 (10)	2.45 (70)
3	1.5172	1.56 (25)	1.54 (30)	1.55 (20)
4	1.4540	1.48 (16)	1.52 (20)	1.49 (5)
5	1.2734	1.29 (10)	1.36 (5)	
6	1.1477	1.16 (8)		

The N_2_ adsorption and desorption isotherm (Fig. S1 and S2†) shows that both N70 and MAS-N70 belonged to type I, with no hysteresis loops. MAS-N70 shows a larger BET surface area of 12.89 m^3^ g^−1^ compared to N70 (4.83 m^3^ g^−1^).

### 3.2. Structural determination and chemical composition of the core–shell@sheet nanostructure

**Fig. 3:**
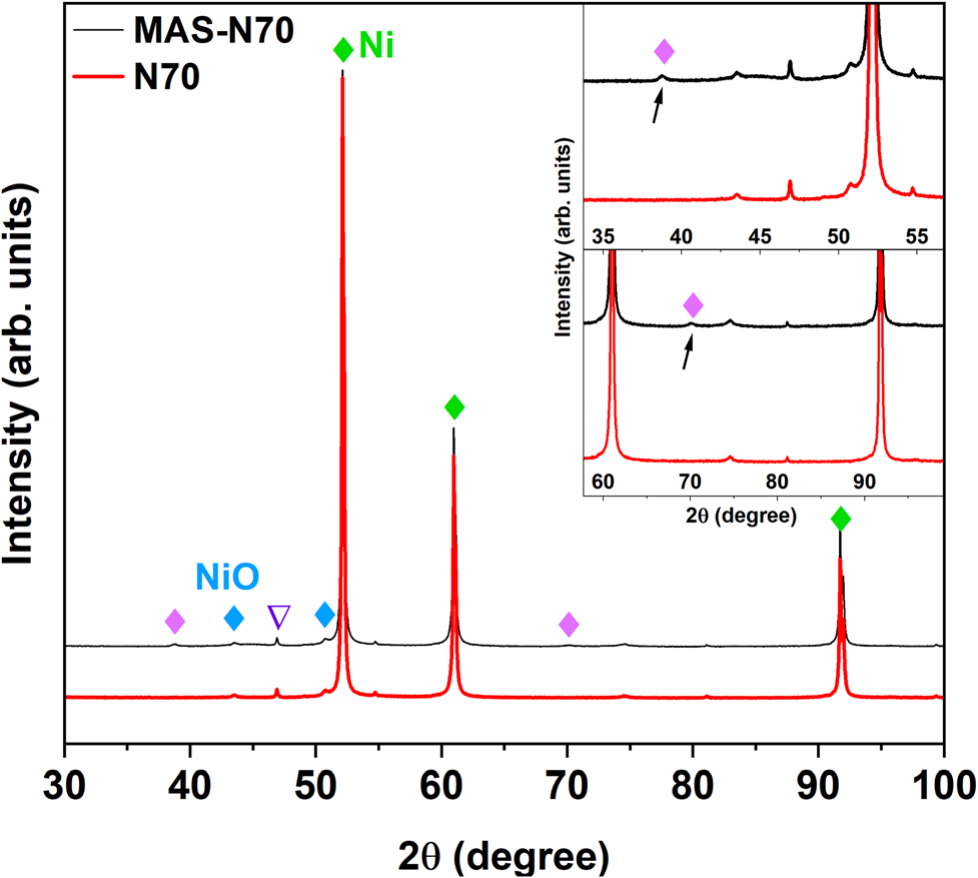
X-ray diffraction pattern of N70 and MAS-N70 (∇; peaks correspond to Co-Kα radiation).

The lab-based XRD patterns for pristine (N70) and MAS-N70 are displayed in Fig. 3. The intense peaks located at 2*θ* ∼ 52.14, 60.99 and 91.73° belong to the (1 1 1), (2 0 0), and (2 2 0) diffraction planes of Ni (JCPDS #04-002-9123), respectively. Furthermore, the peaks observed at 43.52° (1 0 1) and 50.70° (0 1 2) can be attributed to NiO (JCPDS #04-011-2340) suggesting the presence of crystalline Ni and NiO in all the studied samples. Compared to N70, two new diffraction peaks are observed at 2*θ* ∼ 38.76 and 70.11°, which can belong to Ni(OH)_2_, Ni_2_CO_3_(OH)_2_ or Ni_3_CO_3_(OH)_4_.

**Fig. 4:**
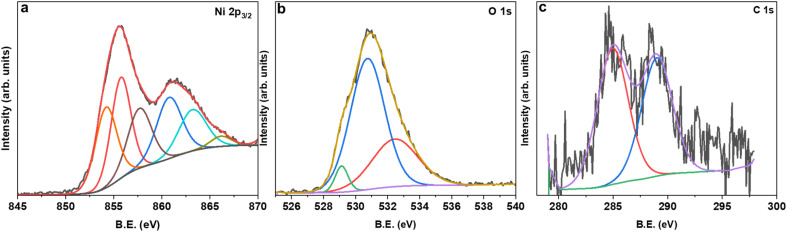
High-resolution fitted XPS spectra of (a) Ni 2p_3/2_, (b) O 1s, and (c) C 1s for MAS-N70.

The XPS survey spectrum for MAS-N70 demonstrates signals corresponding to Ni 2p/3p-, C 1s- and O 1s-core levels. It is noticeable that no additional peaks corresponding to other elements are observed. For detailed investigation on the possible composition of the structure, the XPS data are fitted using Avantage software. Fig. 4 shows the high-resolution fitted XPS spectra of Ni 2p_3/2_, O 1s, and C 1s for MAS-N70. A close look at the Ni 2p_3/2_ spectra (Fig. 4a) reveals three features with a B.E. of ∼854.28, 855.78, and 857.76 eV. The observed peaks corresponding to the B.E. of 854.28 and 855.78 eV can be assigned to nano-NiO.^18^ The main feature is generally assigned to the screened core hole photoemission with the peak at ∼ 854 eV ascribed to local screening from lattice oxygen adjacent to the Ni 2p core hole and the peak at ∼ 855 eV ascribed to non-local screening; however the peak at ∼ 855 eV has also been reported to have a partial contribution from surface states. As per the literature, the XPS peak corresponding to Ni(OH)_2_ appears at a B.E. of 856.3 eV,^19^ thus negating the possibility of NSs made of Ni(OH)_2_. This shows that NSs can possibly be made of Ni_2_CO_3_(OH)_2_ or Ni_3_CO_3_(OH)_4_ and the observed peak at the B.E. of 857.76 eV can possibly be assigned to Ni_2_CO_3_(OH)_2_ or Ni_3_CO_3_(OH)_4_.^20^ Although, the average chemical state of Ni in NiO or Ni_2_CO_3_(OH)_2_ is +2; the B.E. of this signal is determined by the specimen work function, which is a very sensitive property influenced by many variables like surface cleanness, roughness, crystalline phase, or crystal orientation.^21^ For example, Ni in both NiO and Ni(OH)_2_ has an oxidation state of +2; however, their XPS spectra differ completely.^22^Fig. 4b shows the fitted O 1s core level XPS spectrum with peaks at the BE of ∼529.13, 530.76, and 532.49 eV. The peaks at the B.E. of ∼529.13 and 530.76 eV belong to the metal oxide and carbonate, respectively.^23^ The observed peak at the BE of 532.49 eV can be attributed to C–H–O bonding.^24^ The C 1s region (Fig. 4 c) shows peaks at ∼284.8 eV and ∼288.7 eV, which can be attributed to the C–C chemical state and carbonyl group, respectively.^23^

Thus, based on TEM-SAED, XRD and XPS results, a core–shell@sheet structure is considered where the Ni–NiO core–shell structure is wrapped with NSs, which are composed of either Ni_2_CO_3_(OH)_2_ or Ni_3_CO_3_(OH)_4_. To confirm this further, spectroscopic studies are carried out as discussed below.

### 3.3. Spectroscopic determinations

#### Absorption and emission spectroscopy

**Fig. 5:**
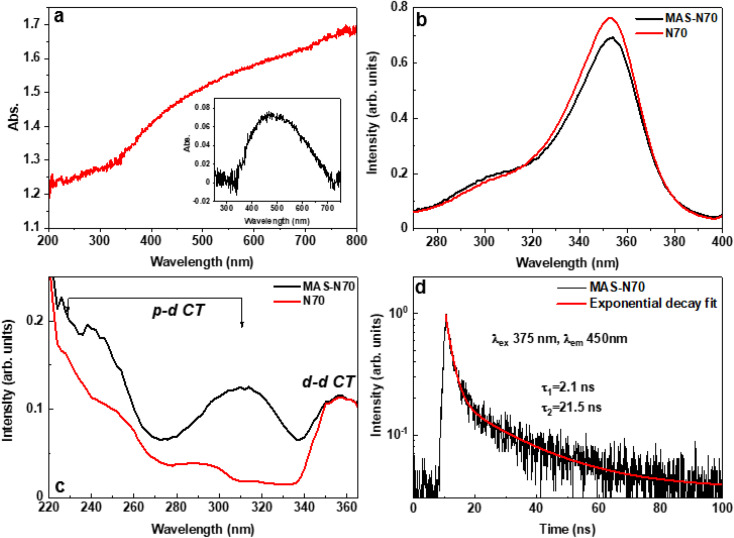
(a) Absorption spectrum, and the inset shows the absorption spectrum with background subtraction, (b) PL spectra under 450 nm (2.8 eV) excitation, (c) excitation spectra monitored with 400 nm (3.1 eV) emission, and (d) time-resolved PL spectra of MAS-N70.

It is well-known that NiO possesses a band gap of ∼3.5–4 eV.^25^ According to Fig. 5a, where the representative UV-vis absorbance spectra for MAS-N70 are shown, no peaks/edges in the wavelength range of 310–355 nm corresponding to NiO are noted. After a careful background subtraction, the UV-vis absorbance spectrum of the MAS-N70 sample shows a broad peak at ∼520 nm compared to the peak at ∼470 nm for N70.^9^ Moreover, the observed peak is broader compared to that noted for N70 (ref. 9) suggesting increased distribution due to the presence of different size NPs. This absorption peak is attributed to the SPR of Ni nanoparticles.

Fig. 5 b–d present the PL results of the studied samples obtained at room temperature. Both samples N70 (pristine) and MAS-N70 exhibit very similar broad emission bands in the blue spectral range peaking at 410 nm and having a shoulder at about 450 nm; therefore, at least two luminescence centers are responsible for the luminescence signal in the samples studied. Based on the literature data we assume that d–d (450 nm) and p–d (410 nm) radiative transitions in NiO have been detected.^26^ Luminescence excitation spectra have been recorded for each of these emissions. The comparison of the excitation spectra for both samples is shown in Fig. 5b and c for the 450 nm and 400 nm emissions, respectively. The strong excitation band peaking at 350 nm (Fig. 5b) well corresponds to the excitation of the d–d transitions and for both samples this band is very similar. On the other hand, the excitation spectra depicted in Fig. 5c are different for the N70 (pristine) and MAS-N70 samples. The excitation spectra in Fig. 5c have the most visible distinguish features at wavelengths shorter than 320 nm. In this excitation energy range the p–p transitions dominate. The excitation curve for the sample MAS-N70 is more intensive and has a more pronounced structure. This fact may indicate that the crystal field around the luminescence center in the sample MAS-N70 is more ordered than in the pristine sample.

The decay kinetics of the 450 nm emission in the sample MAS-N70 are shown in Fig. 5d. The decay curve can be approximated by two exponential fits with the decay times 2.1 and 21.5 ns. The decay curve for the pristine samples is reported in ref. 27 revealing also the two exponential decay law, albeit with slower decay constants 2.3 and 25 ns. The fast component with characteristic time constants of ∼2.1 ns and 2.3 nm for MAS-N70 and pristine N70, respectively, matches with previously reported data for NiO.^26^ On the other hand, the slow decay component of 21.5 ns observed for the MAS-N70 sample (Fig. 5d) is very close to literature data,^26^ while the 25 ns decay constant obtained for the pristine samples is found to be slightly higher than that reported in the literature. This suggests the presence of more trapping states in the pristine sample in comparison with MAS-N70. The origin of such trapping centers is debatable.

#### Raman spectroscopy

**Fig. 6:**
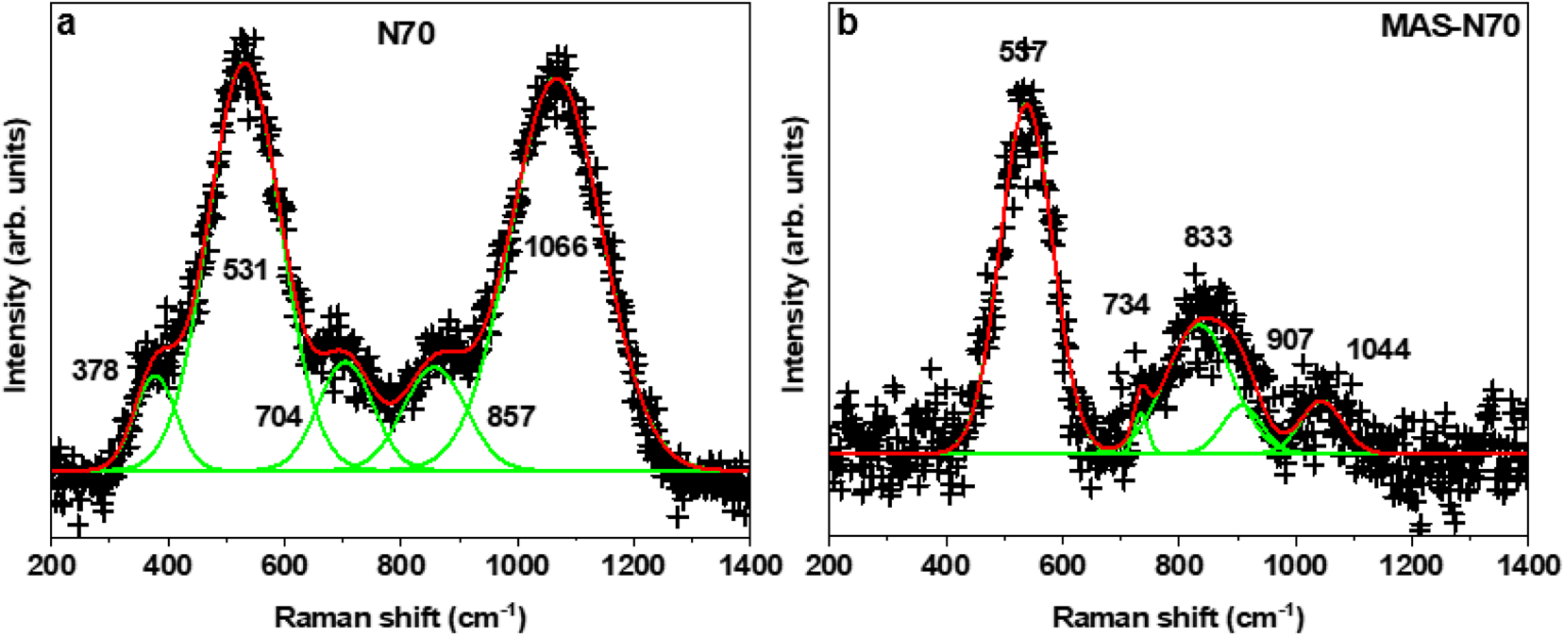
Raman spectrum of N70 (a) and MAS-N70 (b). Solid line (red) is fit to raw data (+).

Fig. 6 shows the Raman spectrum for N70 (Fig. 6a) and MAS-N70 (Fig. 6b). In the Raman spectrum for N70 the optical phonons observed at ∼531, 704, 857, and 1066 cm^−1^ can be assigned to disorder induced one-phonon TO and LO modes, two-phonon TO modes, and TO + LO and 2LO modes of NiO.^28^ Meanwhile phonons observed ∼378 cm^−1^ can be assigned to NiCO_3_.^29^ Note that the disorder-induced one phonon mode at ∼570 cm^−1^ has high intensity due to nanocrystallites of NIO present in the shell. Furthermore, the recorded Raman spectrum for MAS-N70 is quite different from that of N70. The peak corresponding to NiCO_3_ (∼378 cm^−1^) is not observable anymore, showing direct observation of disappearance of NiCO_3_ upon the microwave treatment of N70. The peak corresponding to NiO (∼530 cm^−1^) is still observed due to the presence of the NiO shell in MAS-N70. Apart from that, the other peaks observed can be assigned to Ni_2_CO_3_(OH)_2_.^30^

### 3.4. Photocatalytic hydrogen evolution

**Fig. 7:**
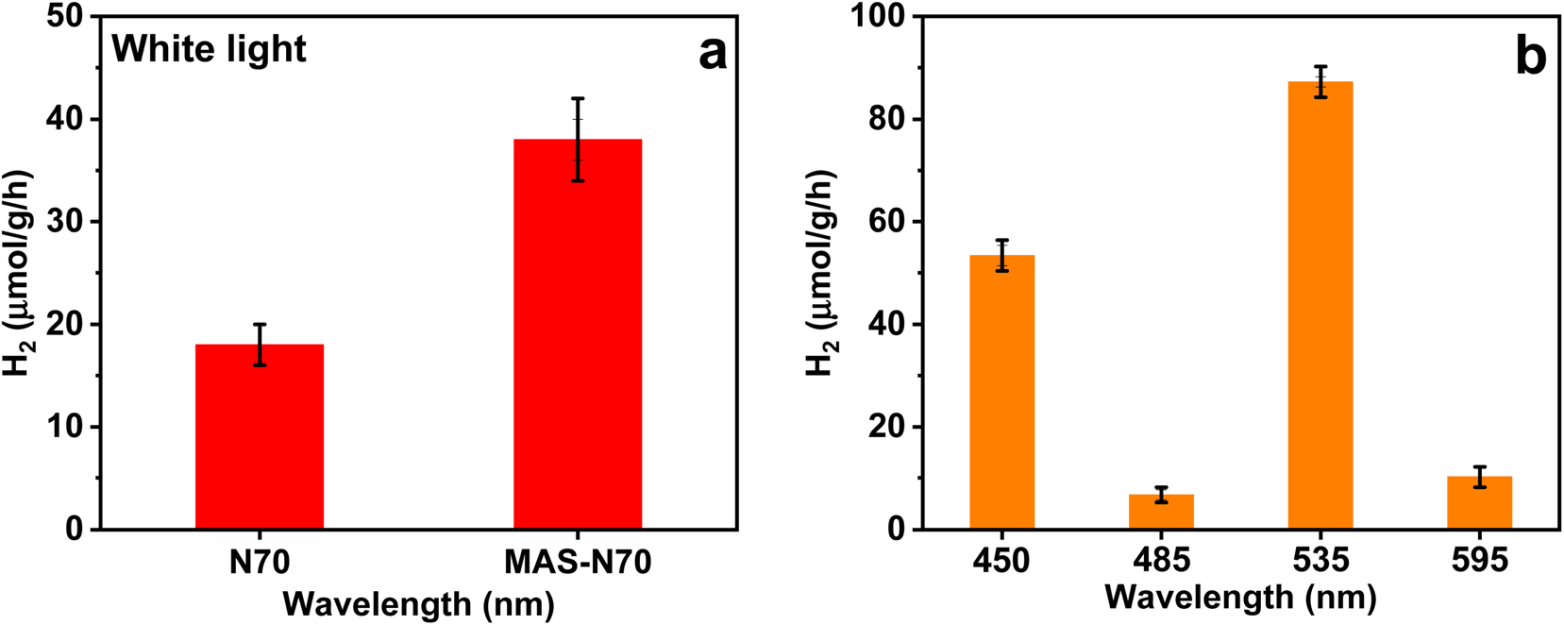
(a) Hydrogen yield for N70 and MAS-N70 under (a) white light and (b) at wavelengths in the range of 450–595 nm.

The results for the water splitting experiment for N70 and MAS-N70 samples under white light illumination are shown in Fig. 7a. With white light irradiation, N70 and MAS-N70 samples show ∼15 μmol g^−1^ h^−1^ and ∼32 μmol g^−1^ h^−1^ HER yields, respectively. Fig. 7b shows the hydrogen production for MAS-N70 in the wavelength range of 420–630 nm giving the highest HER yield (∼87 μmol g^−1^ h^−1^) at a wavelength of 535 nm.

## 4. Microwave assisted flexible nanosheets and corresponding higher HER yield

As per out previous work, based on TEM and XPS analyses, three components, *i.e.*, Ni, NiO, and NiCO_3_, have been observed for N70, while XRD data do not show crystalline NiCO_3_. Thus, it was claimed that Ni NPs are covered with the shell, where the shell contains both crystalline NiO and amorphous NiCO_3_. The pristine sample was further subjected to microwave irradiation, which leads to morphological and structural transitions leading to the final system of Ni–NiO@Ni_2_CO_3_(OH)_2_ as per the following reaction

1 NiCO_3_ → Ni^2+^ + CO_3_^2−^

2 2Ni^2+^ + CO_3_^2−^ + 2OH^−^ → Ni(CO_3_)·Ni(OH)_2_

The irradiation of pristine Ni–NiO/NiCO_3_ core–shell structures leads to breakage of amorphous NiOC_3_. In the presence of water solvent, further interaction between Ni^2+^, CO_3_^2−^, and OH^−^ ions leads to the formation of Ni(CO_3_)·Ni(OH)_2_.

As per the UV-vis and HER results, we notice a broad peak structure at 520 nm and a maximum H_2_ production at 535 nm. Furthermore, no absorption peak corresponding to NiO (3.5–4 eV/310–355 nm) is observed, which indicates that the source with an energy of 2.5 eV cannot excite electrons from NiO, which has a band gap of around 3.5 eV to create electrons and holes for the HER. This clearly indicates the only possible explanation for the observed HER activity is to incorporate the SPR effect of Ni nanoparticles. Incident light causes the SPR effect on the Ni surface, which leads to the generation of hot electrons. Upon illumination with light, Ni NPs generate hot electrons by the SPR effect. Furthermore, since SPR requires a dielectric medium/interface, the NiO shell can act as a dielectric layer to boost the generation of surface plasmons on Ni metal nanoparticles. During the H_2_ production, in the first step, hot charge carriers, *i.e.* electrons (e^−^) and holes (h^+^), are transferred to Ni(CO_3_)·Ni(OH)_2_ NSs *via* the NiO dielectric layer as demonstrated by time-resolved PL. It has also been demonstrated that plasmon relaxation can occur *via* the direct and efficient, simultaneous excitation and transfer of an electron or hole from the metal nanoparticle to the attached semiconductor when a semiconductor is chemically bound to a plasmonic nanoparticle. Moreover, the presence of a thin dielectric layer has enhanced the tunneling of charge carriers from the metal to the semiconductor. Since the surface of the nanosheet → Ni(CO_3_)·Ni(OH)_2_ is in contact with H_2_O, in the second step, h^+^ oxidizes H_2_O and produces H^+^. In the last step of the reaction, Ni(CO_3_)·Ni(OH)_2_ NSs give rise to the HER. As per our recent work, the higher photocatalytic response is due to the favorable electronic structure of NiCO_3_ for the HER with a charge transfer of ∼0.002*e* per atom from the NiCO_3_ surface to the hydrogen molecule when hydrogen is absorbed on the NiCO_3_ surface. The photocatalytic activity can also be enhanced by the incorporation of NiO as a middle layer between Ni and NiCO_3_. Moreover, photoinduced transformation of Ni(OH)_2_ to a defective structure [Ni^0^_*x*_/Ni_1−*x*_(OH)_2_] acts as the real catalytic species of H_2_ photogeneration. Density functional theory (DFT) calculations further indicate that the surface Ni-vacancies (V_Ni_) on the Ni(OH)_2_ nanosheets enhance the adsorption and dissociation of H_2_O molecules to enhance the local proton concentration, while the Ni_0_ clusters behave as H_2_-evolution sites, thereby synergistically promoting the activity of H_2_ photogeneration in alkaline media.^31^

## 5. Conclusions

In conclusion, this study successfully utilized time and power dependent microwave-assisted synthesis to modify pristine visible light active Ni@NiO/NiCO_3_ core–shell photocatalysts, enabling the enhancement of H_2_ yield under visible light. Thorough SEM investigations revealed that the treated photocatalysts, exposed to 800 W for 30 minutes, exhibited a core–shell structure with a Ni–NiO core and flexible nanosheets, whereas TEM-tomography measurements revealed the flexible nature of nanosheets encompassing the Ni–NiO spherical structure. The growth mechanism based on detailed structural and spectroscopy determination suggests that irradiation of pristine Ni–NiO/NiCO_3_ core–shell structures leads to breakage of amorphous NiOC_3_. In the presence of water solvent, further interaction between Ni^2+^, CO_3_^2−^, and OH^−^ ions leads to the formation of Ni(CO_3_)·Ni(OH)_2_. The resulting cross-dimensional core–shell@sheet plasmonic photocatalyst displayed remarkable characteristics for efficient light absorption and photocatalytic applications, demonstrating a twofold increase in the hydrogen evolution reaction yield (40 μmol g^−1^ h^−1^) compared to the pristine core–shell photocatalyst (20 μmol g^−1^ h^−1^) with the highest HER yield of 80 μmol g^−1^ h^−1^ @ 535 nm excitation. These findings highlight the potential of microwave-assisted organic synthesis in developing flexible nanosheets with enhanced performance, while offering valuable insights into their structural and optical properties.

## Conflicts of interest

There are no conflicts to declare.

## Supplementary Material

NA-005-D3NA00583F-s001

NA-005-D3NA00583F-s002
