# Supplementary material for: Flexible nanosheets for plasmonic photocatalysis: microwave-assisted organic synthesis of Ni–NiO@Ni2CO3(OH)2 core–shell@sheet hybrid nanostructures
Source: Nanoscale Adv. 2023 Nov 3;5(24):6935–43. doi: 10.1039/d3na00583f (PMC10697011; doi:10.1039/d3na00583f)
Supplement: NA-005-D3NA00583F-s001 [file NA-005-D3NA00583F-s001.pdf]

Electronic Supplementary Material (ESI) for Nanoscale Advances.
This journal is © The Royal Society of Chemistry 2023

Supplementary information

Flexible Nanosheets for Plasmonic Photocatalysis: Microwave-Assisted Organic Synthesis of Ni-NiO@$Ni_2CO_3(OH)_2$ Core-Shell@Sheet Hybrid Nanostructure

Ekta Rani[1], Parisa Talebi[1], Terhi Pulkkinen[1], Vladimir Pankratov[2], Harishchandra Singh[1*]

[1]*Nano and Molecular Systems Research Unit, University of Oulu, FIN-90014, Finland*
[2] *Institute of Solid-State Physics, University of Latvia, 8 Kengaraga iela, 1063 Riga, Latvia*

*Corresponding author: Harishchandra.Singh@oulu.fi

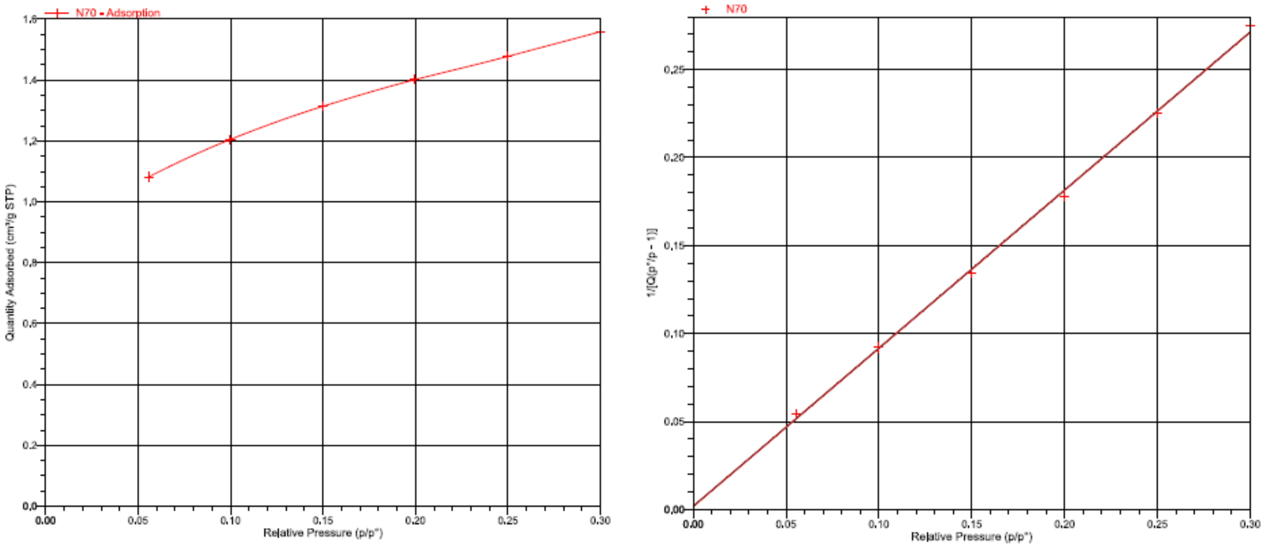

Fig. S1 (a) N_2 adsorption–desorption isotherm and (b) BET surface area plot of N70.

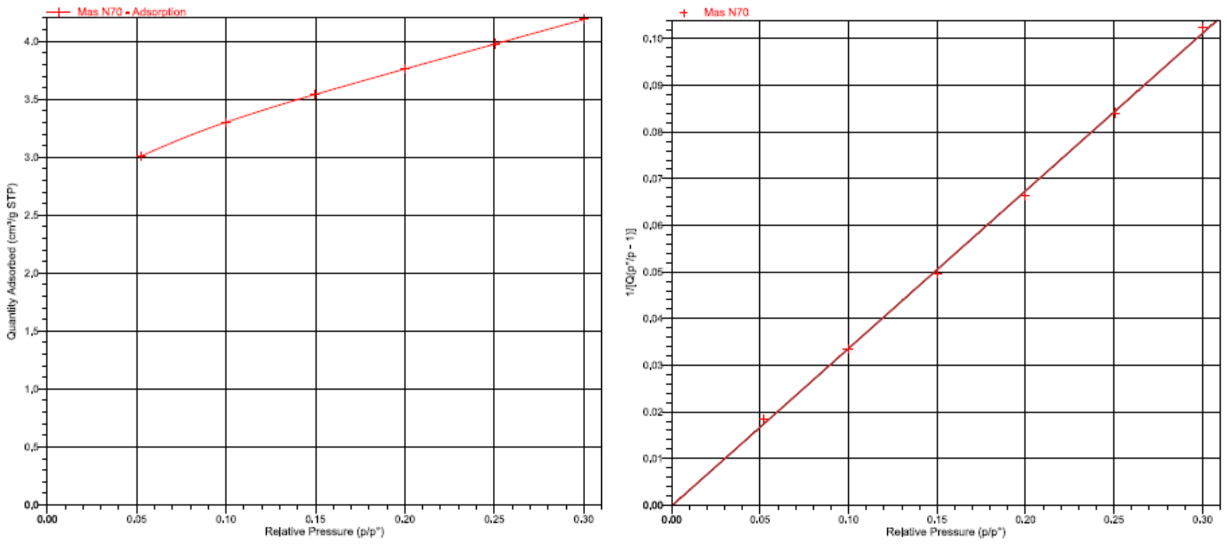

Fig. S2 (a) N_2 adsorption–desorption isotherm and (b) BET surface area plot of MAS-N70.
